# Bioelectric cues from piezoelectric materials in stem-cell adhesion and migration

**DOI:** 10.3389/fcell.2025.1707436

**Published:** 2025-11-06

**Authors:** Ning Chen, Yangyang Su, Renliang Zhao, Xiangtian Deng

**Affiliations:** 1 Nutrition, Food Science and Technology Programme, School of Life Sciences, The Chinese University of Hong Kong, Hong KongSAR, China; 2 West China Clinical Medical College of Sichuan University, Chengdu, China; 3 Department of Orthopedics surgery, Trauma medical center, West China Hospital, Sichuan University, Chengdu, China

**Keywords:** piezoelectric biomaterials, stem cells, cell adhesion, cell migration, tissue regeneration, bioelectric stimulation

## Abstract

Stem cell adhesion and migration are fundamental processes in tissue regeneration and repair; however, their efficiency *in vivo* is often limited by the complexity of the microenvironment. Endogenous bioelectrical cues, such as electric fields present during development and wound healing, play a critical role in guiding these cellular behaviors. Piezoelectric biomaterials, which can convert mechanical stimuli into electrical signals, have recently emerged as promising platforms for recapitulating these bioelectric cues without the need for external power sources. In this mini-review, we summarize the recent advances in the use of piezoelectric scaffolds to modulate stem cell adhesion and migration. We highlight the underlying mechanisms, including integrin/focal adhesion kinase activation, calcium signaling, and electrotaxis, which mediate enhanced adhesion, focal adhesion maturation, and directed cell migration. Representative applications in bone, cartilage, nerve, and muscle tissue engineering are discussed, with an emphasis on how piezoelectric scaffolds improve regeneration by providing dynamic and self-sustained electrical stimulation. Finally, we outline the major challenges, such as balancing piezoelectric output with biocompatibility, controlling *in vivo* stimulation parameters, and elucidating precise sensing mechanisms, and propose future directions for clinical translation. By integrating insights from materials science, mechanobiology, and regenerative medicine, piezoelectric biomaterials hold strong potential as next-generation smart scaffolds for orchestrating stem cell behavior and accelerating functional tissue repair.

## 1 Introduction

Cell adhesion and migration are crucial biological processes for tissue regeneration and form the basis of cellular activities necessary for healing and repair. These processes are dynamic and coordinated, initiating the engraftment, survival, and differentiation of stem cells (Ridley et al., 2003; Devreotes and Horwitz, 2015). The regulation of these phenomena is complex, influenced by numerous factors, including biochemical signals such as growth factors and cytokines (Brizzi et al., 2012; Devreotes and Horwitz, 2015), as well as physical stimuli like mechanical stress, substrate stiffness (Engler et al., 2006; Wozniak and Chen, 2008), and bioelectric fields (Levin, 2021). Among these, endogenous electric fields are particularly noteworthy for their significant role in directing cell migration during critical biological events, including embryonic development, wound healing, and bone repair (Zhao et al., 2006; Zhao, 2008; Griffin and Bayat, 2025). Strategically harnessing these bioelectrical cues offers an innovative approach to enhance regenerative outcomes across a wide range of therapeutic applications (Levin, 2021; Kim et al., 2024).

Piezoelectric biomaterials represent a fascinating class of materials with the unique capability to convert mechanical deformations into localized electric potentials (Mokhtari et al., 2021; Deng et al., 2025). This property effectively simulates the bioelectric stimuli that cells encounter in their natural microenvironments (Vinikoor et al., 2023; Huang et al., 2025). Traditional piezoelectric materials, such as inorganic crystals like barium titanate (BaTiO_3_) and zinc oxide (ZnO), are known for their high piezoelectric coefficients and are commonly used in applications requiring precise electrical responses (Liu et al., 2025). In contrast, organic polymers, including polyvinylidene fluoride (PVDF) and poly (L-lactic acid) (PLLA), provide enhanced flexibility and processability, making them particularly suitable for biomedical applications (Mohammadpourfazeli et al., 2023; Ahbab et al., 2025; Schönlein et al., 2025). When engineered into scaffolds, these piezoelectric materials can generate localized and dynamic electrical signals in response to mechanical loading, cell traction forces, or external ultrasound activation (Cafarelli et al., 2021; Alvarez-Lorenzo et al., 2023). This self-powered electromechanical stimulation offers distinct advantages over conventional inert scaffolds, as it more accurately replicates the dynamic extracellular matrix (ECM) microenvironment crucial for supporting various cellular activities (Ricotti et al., 2024; Zaszczyńska et al., 2024).

This mini-review aims to elucidate the complex mechanisms by which piezoelectric biomaterials influence stem cell adhesion and migration, and explore the implications of these effects within the broader context of tissue regeneration. We will begin with an overview of the fundamental principles underlying piezoelectric biomaterials, detailing their structural characteristics, functional properties, and the mechanisms by which they generate electrical signals in response to mechanical stimuli (Bai et al., 2024; Ni et al., 2025; Wang et al., 2025). A comprehensive understanding of these principles is vital for the rational design and effective application of these materials in regenerative medicine applications. Building on this foundational knowledge, we will investigate the cellular mechanisms governing adhesion and migration, summarizing recent research findings that demonstrate how piezoelectric stimulation can enhance adhesion strength, accelerate focal adhesion turnover, and guide the directional migration of stem cells (Zhao, 2008; Bai et al., 2024; Shlapakova et al., 2024).

Furthermore, we will discuss representative applications of piezoelectric biomaterials in tissue engineering, highlighting their potential to improve outcomes in various clinical scenarios. The prospects for the clinical translation of these innovative materials are also examined, emphasizing the necessity for continued research and development to fully realize their therapeutic potential. By bridging the gap between materials science and cellular biology, this review aims to provide insights that could pave the way for novel strategies in regenerative medicine, ultimately contributing to enhanced healing and recovery processes across a range of tissue types.

## 2 Fundamental principles and properties of piezoelectric biomaterials

The principles underlying piezoelectric biomaterials are based on the phenomenon of piezoelectricity, which refers to the capability of certain materials to produce an electric charge when subjected to mechanical stress (Smith and Kar-Narayan, 2021). This effect arises from mechanical deformation that causes the reorientation of dipoles within a non-centrosymmetric lattice structure or along polymer chains, leading to charge separation and the creation of an electric potential difference across the material (Kamel, 2022; Ahbab et al., 2025). This distinctive characteristic has profound implications for various biomedical applications, particularly in the creation of advanced biomaterials that can dynamically interact with biological systems (Ni et al., 2025).

Inorganic ceramics, such as barium Titanate (BaTiO_3_) and **zinc** Oxide (ZnO), are recognized for their strong piezoelectric responses. These materials possess high piezoelectric coefficients, making them efficient in converting mechanical energy into electrical energy (Lay et al., 2021; Wu et al., 2024). However, their advantageous properties are often tempered by inherent brittleness, which can result in mechanical failure under physiological conditions (Kapat et al., 2020). Furthermore, their limited biodegradability presents challenges for long-term use in implantable devices, as they may not integrate effectively with surrounding biological tissues or degrade appropriately over time (Li et al., 2020; Zheng et al., 2022; Bai et al., 2024).

Conversely, piezoelectric polymers such as Polyvinylidene fluoride (PVDF) and Poly (L-lactic acid) (PLLA) offer a promising alternative (Li et al., 2019; Purushothaman et al., 2023). Although these polymers generally exhibit lower piezoelectric coefficients than their inorganic counterparts, they provide significant benefits in terms of flexibility, biocompatibility, and ease of processing (Li et al., 2019; Zaszczyńska et al., 2024). Their inherent flexibility facilitates better integration with soft biological tissues, making them suitable for applications in tissue engineering and regenerative medicine. Additionally, these polymers can be readily fabricated into nanofibers or hydrogels, enhancing their surface area and promoting cellular interactions, which ultimately aids in tissue regeneration (Dai et al., 2022; Fathollahzadeh et al., 2025).

Recent advancements in biomaterials science have led to the creation of hybrid scaffolds that merge the advantageous properties of both polymers and piezoelectric nanoparticles or aligned nanofibers (Ren et al., 2023). These hybrid materials have shown improved electromechanical conversion capabilities, which can be utilized to stimulate cellular activities and enhance tissue healing (Ricotti et al., 2024; Wu et al., 2024). The incorporation of piezoelectric nanoparticles into polymer matrices not only boosts the piezoelectric response but also preserves the flexibility and biocompatibility of the scaffold, establishing a versatile platform for various biomedical applications (Fathollahzadeh et al., 2025).

One of the most intriguing features of piezoelectric biomaterials is their ability to be activated by physiological movements, such as joint loading or the rhythmic contractions of the heart (Liu et al., 2022). This capability enables the generation of bioelectrical cues in response to the body’s natural movements, which can be essential for promoting cellular responses and facilitating tissue regeneration (Alvarez-Lorenzo et al., 2023). Moreover, these materials can also be stimulated by non-invasive external stimuli, such as ultrasound, allowing researchers and clinicians to control the spatiotemporal delivery of electrical signals *in vivo* (Cafarelli et al., 2021; Ricotti et al., 2024). This characteristic paves the way for new therapeutic interventions, enabling targeted treatments that can adapt to the dynamic nature of the biological systems.

In summary, the principles of piezoelectric biomaterials encompass a wide array of materials, each with distinct properties and potential applications. Ongoing research and development in this field are poised to yield innovative solutions to challenges in tissue engineering, regenerative medicine, and beyond, ultimately advancing healthcare technologies (Chen et al., 2024).

## 3 Mechanisms of stem cell adhesion and migration on piezoelectric scaffolds

The mechanisms of stem cell adhesion are crucial for understanding how stem cells interact with their microenvironment, particularly in tissue engineering and regenerative medicine. Adhesion begins with the integrin-mediated recognition of extracellular matrix (ECM) ligands, which is a fundamental process that allows stem cells to anchor themselves to their surroundings (Huttenlocher and Horwitz, 2011; Iwamoto and Calderwood, 2015; Yamaguchi and Knaut, 2022). This initial interaction is followed by the assembly of focal adhesions, which serve as critical junctions linking the cytoskeleton of the cell to the ECM. The formation and maturation of these focal adhesions are essential for maintaining cellular stability and facilitating communication between the cell and its environment (Iwamoto and Calderwood, 2015; Yamaguchi and Knaut, 2022).

Recent advancements in materials science have introduced piezoelectric surfaces, which can significantly modulate the adhesion process in various ways. One of the primary effects of these surfaces is the enhancement of the adhesion strength. For instance, polarized polyvinylidene fluoride (PVDF) or poly (L-lactic acid) (PLLA) fibers have been shown to increase the adsorption of adhesion proteins on their surface. This leads to the formation of larger and more mature focal adhesions, which are vital for effective cell anchorage and signaling (Ribeiro et al., 2012; Low et al., 2013; Yamaguchi and Knaut, 2022). The increased surface area and the unique electrical properties of these materials create an environment conducive to robust cell adhesion.

Another critical mechanism by which piezoelectric surfaces influence stem cell adhesion is by modulating calcium influx. The electrical potentials generated at the scaffold surface can activate voltage-gated calcium channels, resulting in intracellular calcium transients (Zaszczyńska et al., 2024; Zhang et al., 2024). These calcium signals play a pivotal role in promoting actin polymerization, which is essential for stabilizing adhesion complexes. The dynamic interplay between calcium signaling and cytoskeletal rearrangement underscores the importance of electrical cues in regulating stem cell behavior (Tsai et al., 2015; Lehne et al., 2022).

Furthermore, the activation of focal adhesion kinase (FAK) and the subsequent phosphorylation events are significantly enhanced when stem cells are cultured on piezoelectric scaffolds. This elevated phosphorylation of FAK leads to the activation of downstream signaling pathways, including the mitogen-activated protein kinase (MAPK) and phosphoinositide 3-kinase (PI3K)/Akt pathways. These signaling cascades are crucial for mediating various cellular responses, including proliferation, survival, and differentiation (Velling et al., 2004; Heng et al., 2021; Tan et al., 2023; Katoh, 2024). Through these interconnected mechanisms, piezoelectric stimulation can establish a positive feedback loop. Specifically, stronger cell adhesion leads to greater cellular traction forces that further deform the piezoelectric scaffold. This deformation generates additional electrical cues, which in turn reinforce cellular adhesion (Liu et al., 2021; Xia et al., 2022; Shlapakova et al., 2024; Zaszczyńska et al., 2024).

In addition to adhesion, the mechanisms of directed migration are vital for stem cell functionality. Cell migration is a complex process that requires the establishment of polarity, extension of lamellipodia, formation of new adhesions, and detachment at the rear end (Ridley et al., 2003; Devreotes and Horwitz, 2015). Piezoelectric scaffolds significantly influence this migratory behavior by providing localized electric fields that mimic the phenomenon of electrotaxis (Xia et al., 2022; Alvarez-Lorenzo et al., 2023; Zaszczyńska et al., 2024). One of the key mechanisms involved is the directional bias induced by the voltage gradients along the aligned piezoelectric fibers. This gradient guides mesenchymal stem cells (MSCs) to preferentially migrate toward the cathode, thereby enhancing their migratory efficiency (Banks et al., 2015; Tai et al., 2018; Shlapakova et al., 2024).

Moreover, piezoelectric stimulation increases the migration speed by accelerating adhesion turnover. This results in shorter migration cycles and enhanced net displacement, allowing stem cells to reach target sites more effectively (Xiang et al., 2023). The polarization of signaling molecules, such as PI3K, calcium channels, and integrins, to the cathodal side of the cell further strengthens the front-rear polarity necessary for directed movement (Zhao et al., 2006; Lin et al., 2017; Tsai et al., 2020). Additionally, piezoelectric stimulation can induce cells to secrete chemokines, such as transforming growth factor-beta 1 (TGF-β1), which indirectly amplifies the recruitment of neighboring cells, enhancing the overall regenerative response (Vinikoor et al., 2023; Ricotti et al., 2024).

In summary, piezoelectric scaffolds provide a multifaceted approach to enhance stem cell adhesion and migration, ultimately improving regenerative outcomes in tissue engineering applications (Table 1). The interplay between electrical cues, biochemical signals, and mechanical forces creates a conducive environment for stem cell behavior, paving the way for innovative therapeutic strategies in regenerative medicine.

**TABLE 1:** Representative piezoelectric materials, stimulation modalities, stem cell types, cellular responses, and key signaling pathways are summarized in this review.

Piezoelectric material	Stimulation modality	Stem cell type	Observed cellular response	Key molecular pathways	References
Polarized PVDF film (β-phase)	Static polarization (pre-poled scaffold)	BMSCs (bone marrow MSCs)	**Enhanced adhesion:** Greater cell spreading, stronger attachment and more mature focal adhesions. Thicker, aligned actin fibers formed; improved initial cell anchorage	FAK/Src activation and downstream ERK signaling; Ca^2+^ influx through mechanosensitive ion channels	Tandon et al. (2025)
Electrospun PLLA nanofibers (high Mw)	Dynamic strain (cell-mediated piezo stimulation)	MSCs (mesenchymal stem cells)	**Increased adhesion:** Larger cell spread area and elevated focal adhesion formation. **Osteogenic differentiation upregulated:** higher expression of osteogenic markers (e.g., Runx2, collagen I) on piezoelectric fibers	Integrin clustering with FAK signaling; RhoA/ROCK pathway activation reinforcing the cytoskeleton	Das et al. (2024)
BaTiO3 ceramic nanoparticles (composite scaffold)	Dynamic mechanical loading (*in vivo* movement)	MSCs (osteoprogenitors)	**Accelerated osteogenesis:** Stimulated osteogenic differentiation with increased osteoblast proliferation and extracellular matrix mineralization, leading to faster bone formation	Ca^2+^ influx elevates intracellular calcium signaling; PI3K/Akt pathway activation promoting cell survival and osteogenic gene expression (enhancing bone regeneration)	Wu Q. et al. (2025)
ZnO nanoparticle-infused hydrogel	Ultrasound activation (periodic, wireless)	Neural stem cells (NSCs)	**Directed migration:** Galvanotactic orientation of NSCs toward injury site and enhanced cell infiltration. Supported more robust neuroregeneration (greater new neuron and vessel growth) for improved tissue repair	PI3K/Akt-mediated cell polarity signaling and directed lamellipodia extension; Ca^2+^-dependent signaling cascades (e.g., CaMK activation) triggering migration and regenerative gene programs	Zhang et al. (2025)
P(VDF-TrFE) copolymer	Static polarization/bioreactor culture	MSCs, BM-MSCs, Schwann cells, hiPSC-CMs	Neurite extension and myelination; enhanced osteogenic differentiation; improved cardiac contractility/maturation	Integrin clustering→FAK/Src→ERK; Ca^2+^ influx; PI3K/Akt (via mechanisms described above)	Gryshkov et al. (2021)
P(VDF-TrFE)/BaTiO_3_ composite	Ultrasound activation; polarized surface potential	MSCs	Stronger osteogenic differentiation; accelerated bone defect repair *in vivo*	Ca^2+^ signaling; PI3K/Akt; adhesion-FAK axis	Adolpho et al. (2023)
BiFeO_3_ (BFO) nanofilm (electropositive)	Static positive potential (built-in EF)	MSCs	Increased fibronectin adsorption → more binding sites; enhanced adhesion/migration and osteogenesis; improved osseointegration	FN–integrin engagement → FAK/Src; electrotaxis-assisted recruitment	Sun et al. (2025)
KNN (Na,K-niobate) NP/hydrogel	Dynamic mechanical/ultrasound	BMSCs; NSCs (SCI model)	Osteoblast differentiation (*in vitro*); promoted axon regeneration and cell infiltration *in vivo* spinal cord injury under US activation	Ca^2+^/CaMK axis; metabolic support; PI3K/Akt polarity signaling (per manuscript summary)	Zheng et al. (2025)
PZT ceramic (piezoelectric)	Motion/US-driven microcurrents (nerve conduits)	Neural progenitors/Schwann cells (a concept proposed in the literature)	Wireless neuromodulation for nerve regeneration using movement/US to drive microcurrents (reviewed use in conduits/scaffolds)	Activity through adhesion/ion-channel pathways as summarized in manuscript mechanisms	Tandon et al. (2018)
ZnO/PDMS device-like scaffold	Cyclic bending/stretching	hMSCs → cardiomyogenic constructs	Cardiomyocyte-like maturation/CMs outcomes under cyclic deformation	Mechano-electric Ca^2+^ entry; FAK/ERK coupling (per manuscript mechanisms)	Bhang et al. (2016)
Nylon-11 nanoparticles	Ultrasound activation	Dental pulp stem cells	Induced osteoblast-like differentiation under US-activated piezo signals	Adhesion-FAK and Ca^2+^-dependent osteogenic signaling (per manuscript mechanisms)	Ma et al. (2019)
PHB (piezo polymer)–PANi composite	Ultrasound activation	hBMSCs	Promoted osteoblast differentiation with US-activated piezoelectric response	Integrin/FAK→RUNX2 axis; Ca^2+^/PI3K–Akt (per manuscript mechanisms)	Chernozem et al. (2018)
BNNTs (boron-nitride nanotubes)	Mechanical/US-responsive (reviewed)	NSC/neuronal contexts	Used for non-wired neuronal stimulation and differentiation/regeneration in piezo systems (reported in a review)	Likely Ca^2+^-dependent neuritogenesis with FAK/ERK coupling (per manuscript mechanisms)	Zhu et al. (2024)

## 4 Applications of piezoelectric biomaterials in regenerative medicine

Regenerative medicine has emerged as a transformative field, harnessing the body’s innate healing capabilities to restore function and repair damaged tissues (Mao and Mooney, 2015). Among the various tissues that can be regenerated, bone, cartilage, tendon, neural tissue, skeletal muscle, and cardiac tissue have garnered significant attention due to their complex structures and vital roles in overall health (Alvarez-Lorenzo et al., 2023). The integration of piezoelectric materials into regenerative strategies has opened new avenues for enhancing tissue repair and regeneration (Ahn and Grodzinsky, 2009; Ni et al., 2025).

Bone is a dynamic tissue characterized by its ability to remodel and heal itself, a process that is significantly influenced by mechanical stress (Carter et al., 2021). The inherent piezoelectric properties of bone, primarily attributed to collagen polarization under mechanical load, have inspired the development of piezoelectric scaffolds for bone regeneration. These scaffolds aim to recreate the electrical microenvironments that are often disrupted in bone defects (Kwon and Cho, 2022; Zaszczyńska et al., 2024; Ni et al., 2025). Notably, polarized polyvinylidene fluoride (PVDF) membranes and barium titanate/poly (L-lactic acid) (BaTiO_3_/PLLA) composites have demonstrated remarkable efficacy in accelerating bone healing. The application of these piezoelectric scaffolds has been demonstrated to stimulate osteogenic differentiation and enhance matrix deposition, resulting in improved bone regeneration (Zhou et al., 2016; Dai et al., 2022; Zuchuat et al., 2023). In various animal studies, the implantation of these scaffolds has led to complete closure of calvarial defects and significant regeneration of the osteochondral interface, highlighting their potential for clinical applications in orthopedic surgery (Wu et al., 2022).

Cartilage, being avascular and lacking intrinsic electrical activity, presents unique challenges in regeneration. However, mechanical loading can induce streaming potentials that may be harnessed for therapeutic purposes (Becher et al., 2015; Farooqi et al., 2018). Recent advancements in injectable piezoelectric hydrogels, activated by ultrasound, have shown promise in promoting mesenchymal stem cell (MSC) chondrogenesis and the secretion of transforming growth factor-beta 1 (TGF-β1). This innovative approach has led to the regeneration of hyaline-like cartilage in osteoarthritis models, demonstrating the potential of piezoelectric materials in cartilage repair (Kwon et al., 2016; Vinikoor et al., 2023). Similarly, tendon injuries, which often result in impaired function and pain, can benefit from piezoelectric scaffolds that incorporate these fibers. These scaffolds have been shown to improve collagen alignment and tensile strength, thereby enhancing the repair process and restoring the mechanical integrity of the tendon (Fernandez-Yague et al., 2021; Wu W. et al., 2025).

The intricate architecture of neural tissues necessitates a careful approach to regeneration, particularly because of their sensitivity to electrical cues. Piezoelectric hydrogels containing potassium sodium niobate (KNN) nanoparticles or PVDF nanofibers have been utilized in the context of spinal cord injuries and peripheral nerve repair. Upon ultrasound activation, these scaffolds generate microcurrents that facilitate neural progenitor cell migration, promote axonal outgrowth, and support remyelination. Such enhancements have been linked to functional recovery in rodent models, underscoring the potential of piezoelectric materials in neural tissue engineering (Gryshkov et al., 2021; Tai et al., 2023; Shi et al., 2025).

The regeneration of skeletal and cardiac muscle tissues is critically dependent on rhythmic electrical stimulation, which is essential for their maturation and functional recovery (Ronaldson-Bouchard et al., 2018; Mueller et al., 2020). Piezoelectric scaffolds have been investigated as innovative solutions for providing wireless electrical stimulation (Cafarelli et al., 2021; Ricotti et al., 2024). For example, PVDF-TrFE membranes, when coupled with ultrasound, can generate pulsed electrical signals that synchronize cardiomyocyte contraction *in vitro* (Bryan et al., 2023; Westphal et al., 2023). This concept extends to the development of biodegradable piezoelectric films that could be applied to infarcted myocardium, harnessing thoracic movements to deliver therapeutic stimulation without the need for external leads or batteries (Li et al., 2020; Bai et al., 2024; Chen et al., 2024; Schönlein et al., 2025). Such advancements could revolutionize cardiac repair strategies, offering a more integrated and less invasive approach to treatment than the current strategies.

In conclusion, the integration of piezoelectric materials into regenerative medicine represents a promising frontier with applications spanning bone, cartilage, tendon, neural, skeletal muscle, and cardiac tissues. The ability to harness electrical cues for tissue regeneration not only enhances the healing process but also paves the way for innovative therapeutic strategies that could significantly improve patient outcomes in the future.

## 5 Challenges and future perspectives

Material Design: A high piezoelectric output often leads to a reduction in biocompatibility, particularly in ceramic materials. Future research should prioritize the development of biodegradable composites and bio-derived piezoelectric molecules that effectively balance performance and safety.

*In Vivo* Control: Currently, the electrical output relies on patient activity or external stimuli. Therefore, it is crucial to develop responsive systems that enable precise spatiotemporal control of stimulation.

Mechanistic Understanding: The mechanisms by which cells detect piezoelectric signals remain poorly understood. Potential mediators, such as integrins, calcium channels, and mechanosensitive Piezo ion channels, require further investigation.

Integration with Multimodal Therapies: Future scaffolds should integrate piezoelectric stimulation with drug delivery, magnetothermal regulation, or optogenetics to replicate the complex regenerative environment more accurately.

Clinical Translation: Before piezoelectric scaffolds can be used in clinical trials, long-term safety, degradation products, and regulatory approval must be thoroughly addressed.

## 6 Conclusion

Piezoelectric biomaterials offer a unique strategy for delivering dynamic, self-sustained electrical stimulation to influence stem cell adhesion and migration. By replicating the bioelectric signals inherent in natural regeneration, these advanced scaffolds enhance cellular interactions, facilitate directional migration, and expedite tissue repair across various organ systems. Ongoing advancements in material design, mechanistic understanding, and multimodal integration are expected to address current limitations and propel piezoelectric scaffolds towards clinical application. As research converges at the intersection of materials science, mechanobiology, and regenerative medicine, piezoelectric biomaterials are poised to transform the paradigm of stem cell–based therapies.
